# Prognostic performance of the Rapid Emergency Medicine Score (REMS) and Worthing Physiological Scoring system (WPS) in emergency department

**DOI:** 10.1186/s12245-015-0066-3

**Published:** 2015-06-04

**Authors:** Duc T Ha, Tam Q Dang, Ngoc V Tran, Nhi Y Vo, Nguyen D Nguyen, Tuan V Nguyen

**Affiliations:** Intensive Care Unit, National Hospital of Can Tho, Can Tho City, Vietnam; Department of Internal Medicine, University of Medicine and Pharmacy in Ho Chi Minh City, Ho Chi Minh City, Vietnam; Can Tho University of Medicine and Pharmacy, Can Tho City, Vietnam; Ton Duc Thang University, Ho Chi Minh City, Vietnam; Garvan Institute of Medical Research, Sydney, Australia; School of Public Health and Community Medicine, University of New South Wales, Sydney, Australia; Centre for Health Technologies, University of Technology, Sydney, Australia

**Keywords:** Prognostic model, Emergency department, Rapid emergency medicine score, Worthing physiological scoring system

## Abstract

**Background:**

The Rapid Emergency Medicine Score (REMS) and Worthing Physiological Scoring system (WPS) have been developed for predicting in-hospital mortality in nonsurgical emergency department (ED) patients. The prognostic performance of the scoring systems in independent populations has not been clear. The aim of the study is to evaluate the prognostic accuracy of REMS and WPS systems in the estimation of 30-day mortality risk among medical patients in ED.

**Methods:**

The study was designed as a prospective investigation, with the setting being the ED of the National Hospital of Can Tho, Vietnam. We enrolled medical patients aged 16+ years who met the study entry criteria. Clinical data were obtained as required for each scoring system. The primary outcome was mortality within 30 days since hospitalization. The association between each scoring system and mortality was assessed by the hazard ratio (HR) of the Cox’s proportional hazard model.

**Results:**

The study involved 1746 patients, average age 65.9 years (SD 17). During the period of follow-up, 172 patients (9.9 %) died. The risk of 30-day mortality was increased by 30 % for each additional REMS unit (HR: 1.28; 95 % confidence interval (CI): 1.23–1.34) and by 60 % for each additional WPS unit (HR: 1.6; 95 % CI: 1.5–1.7). The AUC of the REMS was 0.71 (95 % CI: 0.67–0.76) which was significantly lower than that of the WPS (0.80; 95 % CI: 0.76–0.83).

**Conclusions:**

Both REMS and WPS have good prognostic value in the prediction of death in ED patients. The WPS appeared to have a better prognostic performance than the REMS system.

**Electronic supplementary material:**

The online version of this article (doi:10.1186/s12245-015-0066-3) contains supplementary material, which is available to authorized users.

## Background

The assessment of outcomes among patients admitted to a non-surgical medical emergency department (ED) is a challenging task. This is true because ED patients are characterized by a broad spectrum of illnesses and disease severity. Although clinical judgment by experienced clinicians is highly valuable, the judgment is often unreliable, particularly in patients who are in between the two extremes of very seriously ill and ill [1]. Therefore, a number of algorithms have been developed to identify high-risk patients who might need intensive intervention. These algorithms have also been used as a triage tool for allocating appropriate resources on emergency and post-emergency period. Among the algorithms, the Rapid Emergency Medicine Score (REMS) [2], Rapid Acute Physiology Score (RAPS) [3], Worthing Physiological Scoring system (WPS) [4], Routine Laboratory Data (RLD) [5], and Admission Laboratory Tests (ALT) [6] have been widely used in practice [7].

The REMS (Additional file 1) and WPS (Additional file 2) are relatively simple and highly applicable to ED patients, because the input variables are readily available in most ED patients. Both REMS and WPS scoring systems have been validated on European patients in modern hospital environment [8, 4]. In a validation study, REMS was found to be better than RAPS in terms of predicting in-hospital mortality in ED patients, with the area under the receiver operating characteristic curve (AUC) being 0.74 for REMS and 0.64 for RAPS [8]. Furthermore, WPS was found to have good discriminatory power in mortality prediction, with AUC being 0.74 [4]. Nevertheless, it is not clear whether the prognostic performance of the two scoring systems is similar in ED patients, since there were no head-to-head comparison studies. Moreover, both REMS and WPS have not been studied in developing countries, and as a result, their prognostic performance in Asian patients remains largely unknown.

We hypothesized that in Asian patients the prognostic performance of the REMS and WPS in predicting mortality is comparable. The present study was designed to test the hypothesis by pursuing the following specific aims: (1) to determine the prognostic significance of REMS and WPS in predicting the risk of 30-day mortality in Asian patients admitted to ED and (2) to compare the predictive values of REMS and WPS in the patients.

## Methods

### Setting and patients

The study procedures and protocol were approved by the Scientific Research and Ethics Board of the National Hospital of Can Tho (Vietnam). This was a prospective observational investigation, conducted in the ED of the National Hospital of Can Tho, in the Mekong Delta (Vietnam). The hospital is a tertiary teaching hospital that serves 17 million residents in the Mekong Delta region. On average, the hospital’s ED admits 75 non-surgical patients per day.

We enrolled all medical patients aged 16 years and older. “Medical patients” were defined as those were admitted from non-trauma causes with no surgical indication, except surgery for stroke, upper gastrointestinal bleeding, and primary spontaneous pneumothorax. Patients were excluded from the study if they had at least one of the following conditions: acute coronary syndrome, burns, cardiac arrest before admitting to the hospital or which occurred in the ED with failure of cardiopulmonary resuscitation, snakebite, insect bite or sting, poisoning (drugs, alcohol intoxication, paraquat, insecticides, rodenticides, corrosive substances). These patients were transferred to more specialized hospitals for treatment. Patients under 16 years of age, women in labor, and those declared dead on arrival were also excluded from the study.

Due to ethical requirements, patients could withdraw from study at any time without giving reasons. However, in reality, reasons for withdrawal included non-compliance with therapy, transfer to another hospital for more specialized care, loss of follow-up, and family-initiated discharge.

### Study procedure

All patients who met the inclusion criteria were invited to participate in the study. Written informed consent was obtained from all patients. If patients were unconscious or aged less than 18 years, their close relatives or guardians signed the written informed consent. Unconscious patients who regained the cognitive ability were re-consented within 30 days of hospitalization.

Upon the consent was obtained, data collection was conducted by a trained research worker using a structured data form. Patient characteristics, medical history, and physiological data were obtained as required for each scoring system and inclusion criteria (Additional files 1, 2, and 3). Physiological data were recorded at baseline (i.e., first measurement) by medical or nursing staff. After 30 days from the day of admission, the research worker made a call to a relative or delegated person to obtain information on vital status. Patients who had stayed in hospital for more than 30 days were considered “censored”.

### Outcome measures

The primary outcome of the study was mortality within 30 days since the time of admission to the hospital. Mortality was defined as (a) patients who died in hospital from any cause; (b) family-initiated discharged patients who died either on the way home or within 24 h after the hospital discharge; (c) doctor-initiated discharged patients who died at home. It should be noted that in Vietnamese culture, patients prefer to die at home rather than in hospital. As a result, when a patient is in the terminal stage, the patient or family usually requests for discharge from hospital. The secondary outcome was length of hospital stay, which was determined from the time of hospital admission to the time of discharge.

### Risk factors

The research staff recorded data on pulse, body temperature, blood pressure (automatic blood pressure monitor, OMRON HEALTHCARE Co, Vietnam), breathing rate, peripheral oxygen saturation (NONIN Co, USA), days of therapy from other hospital(s), doses of vasopressor agents, cardiopulmonary resuscitation, mechanical ventilation, comorbidity, and functional status (Additional file 3). In some cases (approximately 5 %) where blood pressure was too low or too high, the automatic blood pressure monitor could not measure, and we manually took the blood pressure measurements.

### Data management and analysis

We used the double data entry approach to enter data. The first data entry was undertaken within a day after hospital admission. The second data entry was done at the end of the study. Data from the two entries were then independently verified to check for potential discrepancies. Any discrepancy was adjudicated with the original patient record.

Data analysis was conducted according to a plan that was set out prior to the data collection. In the first stage, we calculated descriptive statistics (i.e., mean, standard deviation, median with interquartile range, proportion) for each physiological variable for the REMS and WPS systems with stratification by survival status. Categorical variables will be tested by Fisher’s exact test. The distribution of continuous variables was tested by Shapiro-Wilk normality test. The relationship between continuous variables and 30-day mortality will be tested by the Student’s *t* test for normally distributed variables or permutation test for non-normally distributed variables. In the next stage, we used the Cox’s proportional hazards model to evaluate the association between REMS and WPS and mortality risk. The discrimination of each scoring system was assessed by AUC value. The difference between two AUCs was tested by the DeLong method [9]. All statistical analyses were performed with the R Statistical Environment version 3.1.0 [10].

## Results

Between March 13, 2013 and June 1, 2013, we had enrolled 2179 patients into the study. However, 71 patients were excluded from the analysis because they did not meet inclusion/exclusion criteria; 271 patients were discontinued from the study. The reasons for discontinuation were as follows: loss to follow-up (28 patients), family-initiated discharge or transfer to another hospital (243 patients). Among the remaining 1837 patients, there were 91 patients with missing values. Therefore, the data were analyzed from 1746 patients.

The median length of stay was 7 days (IQR: 4 to 10). During the follow-up period, 172 patients died within 30-day after admission, and this represents a mortality rate of 9.9 % (95 % confidence interval (CI): 8.5–11.3 %). The 5-day mortality rate was 5.7 % (95 % CI: 4.6–6.9), and this rate increased progressively with time of hospitalization.

Baseline clinical characteristics of patients stratified by survival status are shown in Table 1. Most of risk factors from REMS and WPS were significant predictors of mortality in univariate analysis (Tables 2 and 3). Among the risk factors, the most prominent factors included breathing rate greater than 49 times per minute or less than 6 times per minute, peripheral oxygen saturation less than 75 %, and coma level with Glasgow coma score less than 5 points or AVPU scale (A: Alert, V: Verbal, P: Pain, U: Unresponsive) different from alert (Additional files 4 and 5). Age was not a clear risk factor.

**Table 1:** Baseline characteristics of 1746 ED patients stratified by survival status

Variable	Alive after 30 days	Deceased within 30 days	*P* value
	*n* (%) or median (Q1, Q3)	*n* (%) or median (Q1, Q3)	
Number of patients	1574	172	
Number of female patients (%)	865 (55.0)	75 (43.6)	0.005
Age (year)	68 (55, 80)	71 (59, 81)	0.219
Pulse (per min)	90 (78, 103)	100 (83, 115)	<0.001
Body temperature (°C)	37.1 (37.0, 37.7)	37.3 (37.0, 38.1)	<0.001
Systolic blood pressure (mmHg)	139 (120, 159)	131 (110, 158)	0.051
Diastolic blood pressure (mmHg)	80 (70, 90)	78 (63, 90)	0.178
Mean arterial pressure (mmHg)	99 (87, 112)	96 (80, 110)	0.081
Breathing rate (per min)	22 (20, 26)	26 (22, 30)	<0.001
Peripheral oxygen saturation (%)	97 (94, 99)	93.5 (87, 97)	<0.001
Days of therapy from other hospital (days)	0 (0, 29)^a^	0 (0, 63)^a^	0.005
Days from illness onset (days)	1 (0, 3)	1 (0, 2)	0.279
Dopamine (μg/kg/min)	0 (0.0, 6.8)^a^	0 (0.0, 10)^a^	<0.001
Norepinephrine (μg/kg/min)	0 (0.0, 2.0)^a^	0 (0.0, 2.8)^a^	0.008
Dobutamine (μg/kg/min)	0 (0.0, 9.8)^a^	0 (0.0, 11.0)^a^	0.198
Length of stay (days)	7 (4, 10)	3 (1, 8)	<0.001
Glasgow coma score	15 (15, 15)	15 (10, 15)	<0.001
AVPU scale			<0.001
Unresponsive to all stimuli	6 (0.4)	11 (6.4)	
Responds to painful stimuli	39 (2.5)	37 (21.5)	
Responds to verbal stimuli	48 (3.0)	14 (8.1)	
Alert	1481 (94.1)	110 (64.0)	
Cardiopulmonary resuscitation	1 (0.1)	3 (1.7)	0.003
Mechanical ventilation	15 (1.0)	25 (14.5)	<0.001
Admitted intensive care unit	33 (2.1)	63 (36.6)	<0.001
Immunocompromised by agent	27 (1.7)	4 (2.3)	0.539
Lymphoma	2 (0.1)	1 (0.6)	0.268
Leukemia or myeloma	8 (0.5)	3 (1.7)	0.086
Cancer	18 (1.1)	5 (2.9)	0.068
Chronic renal failure	66 (4.2)	10 (5.8)	0.323
Chronic respiratory failure	88 (5.6)	19 (11.0)	0.011
Cirrhosis with ascites	44 (2.8)	10 (5.8)	0.037
Heart failure	87 (5.5)	24 (14.0)	<0.001
Diabetes mellitus	530 (33.7)	75 (43.6)	0.011
Functional status			0.013
Independence	1357 (86.2)	137 (79.7)	
Partial dependence	161 (10.2)	21 (12.2)	
Completely dependence	56 (3.6)	14 (8.1)	
REMS score	6 (4, 8)	9 (6, 12)	<0.001
WPS score	2 (1, 4)	5 (3, 6)	<0.001

^a^Median (min, max)

**Table 2:** Association between REMS factors and 30-day mortality risk in ED patients

Variable	Hazard ratio (95 % confidence interval)	*P* value
Body temperature (°C)^a^		
36.0 to 38.4	1.0	
34.0 to 35.9/38.5 to 38.9	1.8 (1.1–3.0)	0.025
30.0 to 31.9/39.0 to 40.9	1.8 (1.2–2.9)	0.009
<30.0/>40.9	4.9 (0.7–35.1)	0.113
Mean arterial pressure (mmHg)^a^		
70 to 109	1.0	
50 to 70/110 to 130	1.2 (0.8–1.7)	0.372
130 to 159	1.9 (1.2–3.1)	0.005
<50/>159	4.7 (2.3–9.7)	<0.001
Pulse (per minute)^a^		
70 to 109	1.0	
55 to 69/110 to 139	1.6 (1.1–2.2)	0.008
40 to 54/140 to 179	4.6 (2.7–7.8)	<0.001
<40/>179	5.9 (0.0–infinitive)	0.992
Breathing rate (per minute)^a^		
12 to 24	1.0	
10 to 11/25 to 34	2.5 (1.8–3.5)	<0.001
35 to 49	5.0 (3.0–8.2)	<0.001
<6/>49	8.3 (2.6–26.2)	<0.001
Peripheral oxygen saturation (%)^a^		
>89	1.0	
86 to 89	3.5 (2.2–5.6)	<0.001
75 to 85	6.1 (3.9–9.5)	<0.001
<75	11.9 (6.4–22.1)	<0.001
Glasgow coma score^a^		
>13	1.0	
11 to 13	4.6 (2.7–7.8)	<0.001
8 to 10	5.7 (3.4–9.5)	<0.001
5 to 7	14.7 (9.1–23.6)	<0.001
<5	26.2 (13.6–50.2)	<0.001
Age^a^		
<45	1.0	
45 to 54	0.9 (0.5–1.8)	0.773
55 to 64	1.1 (0.6–2.0)	0.739
65 to 74	1.1 (0.6–2.1)	0.645
>74	1.4 (0.8–2.3)	0.250
REMS score (+1)	1.28 (1.23–1.34)	<0.001

^a^Classification according to the REMS system

**Table 3:** Association between WPS factors and 30-day mortality risk in ED patients

Variable	Hazard ratio (95 % confidence interval)	*P* value
Breathing rate (per minute)^a^		
<20	1.0	
20–21	1.3 (0.7–2.5)	0.419
>21	2.8 (1.7–4.8)	<0.001
Pulse (per minute)^a^		
<102	1.0	
≥102	1.9 (1.4–2.6)	<0.001
Systolic blood pressure (mmHg)^a^		
>99	1.0	
≤99	2.8 (1.9–4.3)	<0.001
Peripheral oxygen saturation (%)^a^		
96 to 100	1.0	
94 to 95	2.1 (1.4–3.3)	0.001
92 to 93	2.6 (1.5–4.4)	<0.001
<92	5.8 (4.1–8.3)	<0.001
AVPU level^a^		
3	1.0	
0 to 2	7.5 (5.5–10.3)	<0.001
WPS score (+1)	1.6 (1.5–1.7)	<0.001

^a^Classification according to WPS

### Discrimination analysis

**Fig. 1:**
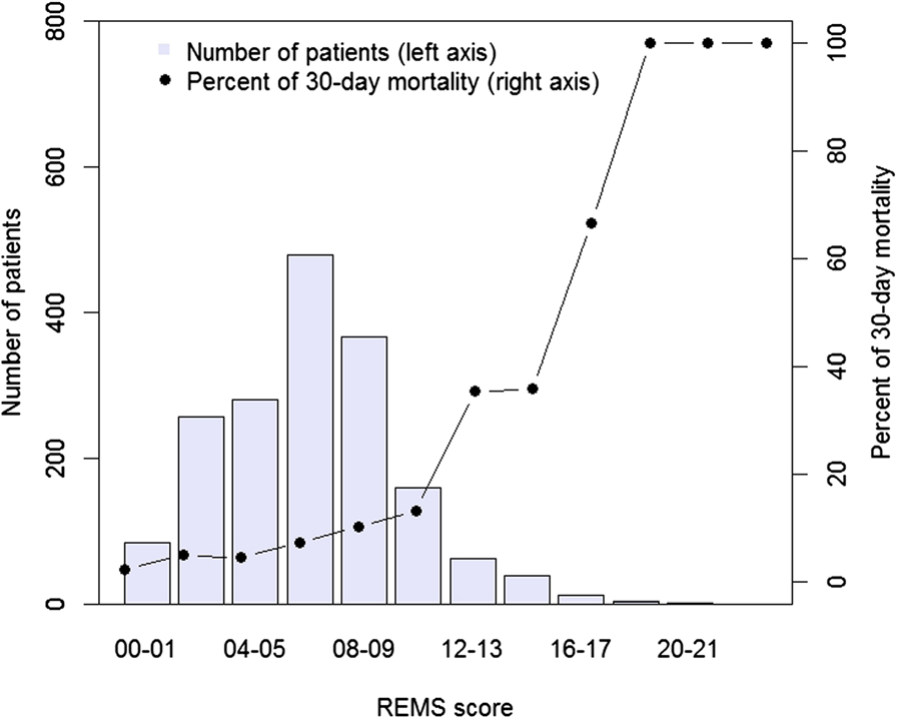
Distribution of the REMS score (*left axis*) and percent of mortality associated with each score (*right axis*)

**Fig. 2:**
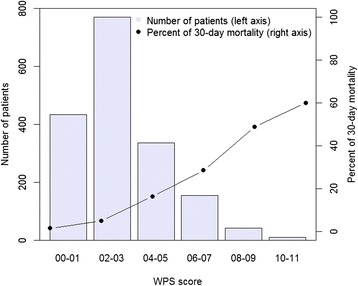
Distribution of WPS score (*left axis*) and percent of mortality associated with each score (*right axis*)

The distribution of REMS scores is shown in Fig. 1. The median REMS score was 6 points (IQR: 5 to 8). Approximately 1 % (*n* = 18) of patients had REMS score ≥16 points, and among these patients the rate of mortality was ~78 % (*n* = 14). Moreover, the median WPS score was 2 points (IQR: 2 to 4). Although there were about 3 % (*n* = 53) of patients with WPS score ≥8 points, the rate of mortality among them was ~51 % (*n* = 27) (Fig. 2). The risk of mortality progressively increased with higher score of REMS and WPS. As expected, the survival group had lower scores than deceased group for REMS (6 vs 9 points, *P* < 0.001) and WPS (2 vs 5 points, *P* < 0.001 (Table 1).

**Fig. 3:**
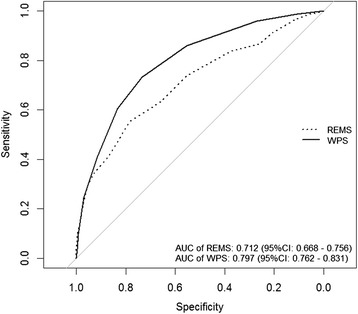
Area under the receiver operating characteristic curves for REMS and WPS in the prediction of 30-day mortality (*P* value <0.001, DeLong’s test for the two correlated receiver operating characteristic curves)

**Fig. 4:**
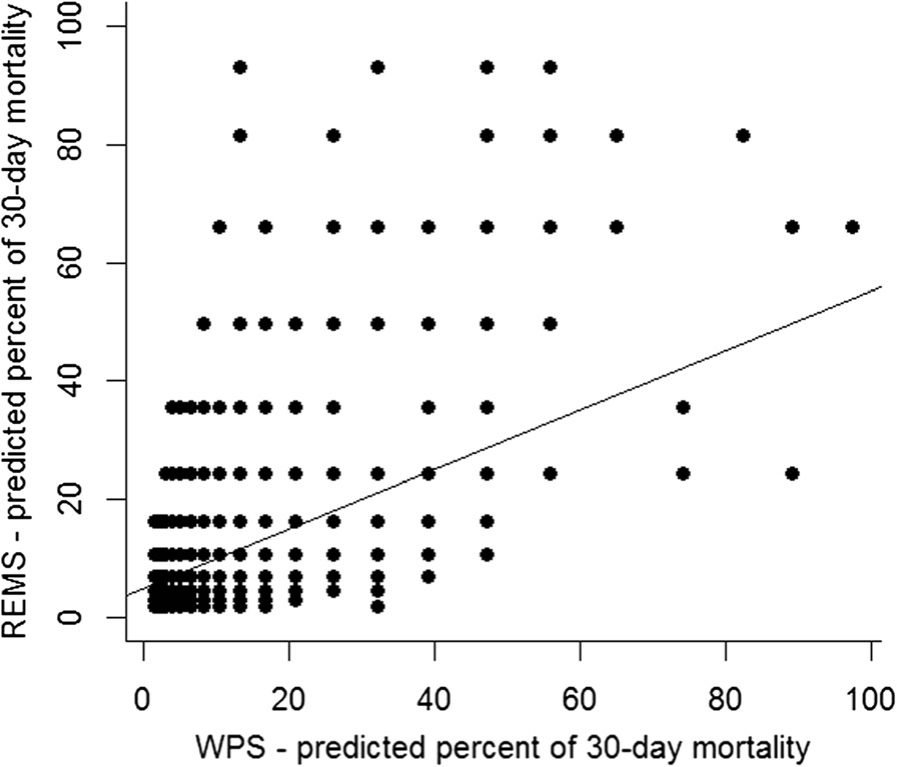
Concordance between *REMS* predicted and *WPS* predicted percent of mortality

In the Cox’s proportional hazards model, each unit increase in the REMS was associated with 30 % increase in the risk of mortality (HR 1.28; 95 % CI: 1.23–1.34), and each unit increase in WPS was associated with a 60 % risk of mortality (HR 1.6; 95 % CI: 1.5–1.7). The area under the receiver operating characteristic curve for REMS and WPS in mortality prediction is shown in Fig. 3. The AUC for WPS (0.797) was significantly greater than that for REMS (0.712). In addition, there was a modest correlation between the predicted risk of mortality by REMS and predicted risk of mortality by WPS (Fig. 4), with the Spearman’s rank correlation being 0.45 (*P* < 0.001).

### Calibration analysis

**Fig. 5:**
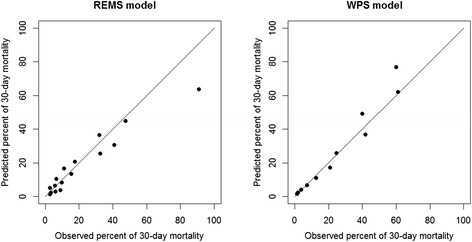
Observed and predicted percent of mortality by *REMS* and *WPS* algorithm

In each scoring system, there was good calibration (i.e., agreement between observed and predicted rate of mortality). However, a close examination of data revealed that REMS tended underestimate the risk of death in high-risk patients. On the other hand, WPS tended to overestimate the risk of death in high-risk patients (Fig. 5).

## Discussion

The assessment of outcome in medical patients is a challenging task, because the diversity and variation in severity of the patient population. The difficulty in the assessment of ED patients is also related to the problem of assigning appropriate weight to each symptom and comorbidity. In that setting, it appears that clinical algorithms with statistical weighting can offer useful prognostic information. In this study, we compared the prognostic performance of two popular algorithms, REMS and WPS, in the prediction of mortality in ED patients. We found that both algorithms have good discrimination and calibration, but the WPS appeared to have better discriminatory power than the REMS system. These findings deserve more elaboration.

It is difficult to compare the present results with previous studies, because there have been no external validation studies for the WPS algorithm. In the original study where REMS was developed, the AUC was 0.852 [2], which was higher than our observed AUC (0.712). We note that in the original study, the mortality rate was 2.4 %, which is much lower than our study’s (9.9 %); the original population was from ED (~56 % hospitalized patients) which is also different from our target population. We also note that the REMS and WPS were designed to predict the risk of in-patient mortality, whereas in our study the outcome was 30-day mortality, and this difference could partly explain the difference in AUC values. It is unlikely that any single prognostic model will perform well for a whole spectrum of seriously ill in ED [11]. However, our study demonstrated that the calibration (e.g., agreement between observed and predicted probability) of the REMS system was very good.

Our result shows that the WPS system had better mortality discrimination than the REMS system. In the initial study [4], the AUC was 0.740, which was lower than the present study’s (0.797). The calibration of WPS was also reasonably good. We note that the relationship between mortality risk and WPS scores was approximately linear, such that patients in the top scores (10–11) had a risk of death over 10 times greater than those in the lowest range (0–1), suggesting a good discrimination.

However, it is interesting to observe that there was a modest correlation between the predicted risk of death by REMS and WPS systems. For a given WPS-predicted risk, there is a high variability in the REMS-predicted risk. Nevertheless, a combination of both REMS and WPS systems did not improve the AUC value over and above the AUC of the WPS alone, suggesting that REMS contributes little to the prediction of mortality by WPS.

The use of REMS or WPS is highly feasible in the ED setting, because the two systems use data that are either readily available from patients at the time of admission or routinely collected in the ED. However, both systems categorize continuous data with distinct groups, and this categorization could be a potential weakness. It is well known that any categorization of continuous data can result in loss of information and loss of statistical power. Thus, we consider that there is room for improvement of the predictive value of the REMS and WPS system.

To our knowledge, this is the first validation of REMS and WPS systems in a head-to-head way and in a developing country hospital. However, the AUC is still not confidently high, suggesting that false positives and false negatives are still a problem. Nevertheless, the use of WPS in conjunction of experienced clinical judgment can help better assess outcome for ED patients.

The present findings have to be considered within the context of strength and limitation of the study. The study was based on a large sample of patients, which allows the delineation of modest effect sizes otherwise not detected by smaller studies. The assessment of outcome and the data collection were rigorously done to ensure the accuracy and reliability of data. However, patients in this study were drawn from a population whose socioeconomic status and cultural values are not necessarily the same with the Caucasian populations whose REMS and WPS were based on. In terms of cultural values, it should be noted that Vietnamese patients prefer to pass away in their home rather than in hospital, and this is the reason for including death occurred at home after 24 h of hospital discharge as an outcome. Although the National Hospital of Can Tho is a major and teaching hospital in Vietnam, its resources and infrastructure are not necessarily as good as in most Western teaching hospitals, and the difference in mortality rates may be expected. In addition, the rate of loss to follow-up (~13 %) could potentially reduce the sensitivity of the scoring systems.

## Conclusions

In summary, we have demonstrated in this study that both REMS and WPS scoring systems have good prognostic value in the prediction of mortality in ED patients in a tertiary teaching hospital in Vietnam. We also found that the WPS has a better discrimination than the REMS system. The application of WPS together with clinical judgment may provide useful information to clinicians in the allocation of resources and treatment of ED patients.

## Additional files

Additional file 1:
**The scoring system for REMS.** This file contains variables and their scoring used in the REMS prognostic model. Additional file 2:
**The scoring system for WPS.** This file contains variables and their scoring used in the WPS prognostic model. Additional file 3:
**Definition of variables and methods of measurement in the study.**
Additional file 4:
**The Glasgow coma score.**
Additional file 5:
**The AVPU scale.**
