# Supplementary material for: Prognostic performance of the Rapid Emergency Medicine Score (REMS) and Worthing Physiological Scoring system (WPS) in emergency department
Source: Int J Emerg Med. 2015 Jun 4;8:18. doi: 10.1186/s12245-015-0066-3 (PMC4457731; doi:10.1186/s12245-015-0066-3)
Supplement: Additional file 1: — The scoring system for REMS. This file contains variables and their scoring used in the REMS prognostic model. [file 12245_2015_66_MOESM1_ESM.docx]

| **Additional file 1. The scoring system for REMS [**[**1**](#_ENREF_1)**]** | | | | | | | |
| --- | --- | --- | --- | --- | --- | --- | --- |
| Variable | Score | | | | | | |
|  | 0 | +1 | +2 | +3 | +4 | +5 | +6 |
| Age (years) | <45 |  | 45–54 | 55-64 |  | 65–74 | >74 |
| Body temperature (^o^C) | 36-38.4 | 38.5–38.9  34–35.9 | 32–33.9 | 39–40.9  30–31.9 | >40.9  <30 |  |  |
| Mean arterial pressure (mmHg) | 70-109 |  | 110 – 129  50 - 69 | 130-159 | >159  <50 |  |  |
| Pulse (per min) | 70-109 |  | 110 – 139  55 - 69 | 140–179  40-54 | >179  <40 |  |  |
| Breathing rate (per min) | 12–24 | 25–34,  10 – 11 | 6-9 | 35-49 | >49  <6 |  |  |
| Peripheral oxygen saturation (%) | >89 | 86-89 |  | 75-85 | <75 |  |  |
| Glasgow coma score | >13 | 11-13 | 8 - 10 | 5-7 | <5 |  |  |

**Reference**

1. Olsson T, Terent A, Lind L (2004) Rapid Emergency Medicine score: a new prognostic tool for in-hospital mortality in nonsurgical emergency department patients. Journal of internal medicine 255: 579-587.
