# Supplementary material for: Prognostic performance of the Rapid Emergency Medicine Score (REMS) and Worthing Physiological Scoring system (WPS) in emergency department
Source: Int J Emerg Med. 2015 Jun 4;8:18. doi: 10.1186/s12245-015-0066-3 (PMC4457731; doi:10.1186/s12245-015-0066-3)
Supplement: Additional file 2: — The scoring system for WPS. This file contains variables and their scoring used in the WPS prognostic model. [file 12245_2015_66_MOESM2_ESM.docx]

| **Additional file 2**. **The scoring system for WPS [**[**1**](#_ENREF_1)**]** | | | | |
| --- | --- | --- | --- | --- |
|  | Score |  |  |  |
|  | 0 | +1 | +2 | +3 |
| Breathing rate (per min) | ≤19 | 20 – 21 | ≥22 |  |
| Pulse (per min) | ≤101 | ≥102 |  |  |
| Systolic blood pressure (mmHg) | ≥100 |  | ≤99 |  |
| Body temperature (^o^C) | ≥35.3 |  |  | <35.3 |
| Peripheral oxygen saturation (%) | 96 – 100 | 94 – 95 | 92 – 93 | <92 |
| AVPU scale | Alert |  |  | Other |

**Reference**

1. Duckitt RW, Buxton-Thomas R, Walker J, Cheek E, Bewick V, et al. (2007) Worthing physiological scoring system: derivation and validation of a physiological early-warning system for medical admissions. An observational, population-based single-centre study. British journal of anaesthesia 98: 769-774.
