# Supplementary material for: Prognostic performance of the Rapid Emergency Medicine Score (REMS) and Worthing Physiological Scoring system (WPS) in emergency department
Source: Int J Emerg Med. 2015 Jun 4;8:18. doi: 10.1186/s12245-015-0066-3 (PMC4457731; doi:10.1186/s12245-015-0066-3)
Supplement: Additional file 3: — Definition of variables and methods of measurement in the study. [file 12245_2015_66_MOESM3_ESM.docx]

| **Additional file 3**. **Variables and method of measurement.** | |
| --- | --- |
| **Variable** | **Definition and method of measurement** |
| Pulse | Enrolled within 15 minutes from admitting to ED. Pulse was counted for a minute. If there was no pulse, pulse was recorded as zero. Locations of taking pulse were radial artery, brachial artery, carotid artery, femoral artery. |
| Body temperature | Maximal temperature was recorded at ED. Unit of measurement was Celsius degree. Body temperature was taken in axilla and plus 0.5 Celsius degree. |
| Blood pressure | Enrolled within 15 minutes from admitting to ED. |
| Mean arterial pressure | Defined by formula: $mean arterial pressure=\frac{1}{3}*systolic blood pressure+\frac{2}{3}*diastolic blood pressure$ |
| Breathing rate | Enrolled within 15 minutes from admitting to ED. Breathing rate was counted for a minute. If the patient was ventilated through tracheal tube, self-breathing rate was recorded. If the patient did not breathe within 15 seconds, breathing rate was recorded as zero. |
| Peripheral oxygen saturation | Enrolled within 15 minutes from admitting to ED. Peripheral oxygen saturation (SpO_2_) was checked for a minute with room air. |
| Days of therapy from other hospital | Days in which the patient was treated from other hospital before transferring to ED. |
| Days from illness onset | The days that the patient’s health was worse than baseline (not including the days of therapy from other hospital). The change from baseline could be sudden or gradual. |
| Doses of dopamin, norepinephrine, and dobutamine | Doses of vasopressor agents were enrolled at the time of transferring patient to another department. Dose was recorded only being used for more than an hour[[1](#_ENREF_1)]. Patient’s weight was determined by asking patient or by a scale. |
| Glasgow coma score | Maximal Glasgow coma score which was recorded at ED. |
| AVPU scale | It was the best AVPU level to be recorded at ED. |
| Cardiopulmonary resuscitation | Cardiopulmonary resuscitation happened at ED or other hospital. |
| Mechanical ventilation | Invasive ventilation at ED. |
| Admitted intensive care unit | Patient admitted to intensive care unit with anticipating. |
| Immunocompromised by agent | Medical history of using cytotoxic agents within 3 months [[2](#_ENREF_2)], or corticosteroid equivalent to 1mg/kg/day prednisone for greater than 1 month during 3 months preceding ED admission [[3](#_ENREF_3)]. |
| Lymphoma, leukemia or myeloma, and other cancer | History of diagnosis with lymphoma, leukemia or myeloma, or other cancer. |
| Chronic renal failure | History of indication of routine hemodialysis or peritoneal dialysis due to chronic renal failure. |
| Chronic respiratory failure | History of diagnosis with chronic respiratory disease and dyspnea with activities of daily living or at rest[[4](#_ENREF_4)]. |
| Cirrhosis with ascites | Evidence of ascites, prothrombin time less than 51%, and coarse hepatic parenchymal echotexture appearing on ultrasonography [[5](#_ENREF_5)]. |
| Heart failure | Heart failure was defined according to the criteria described in [[6](#_ENREF_6)]. |
| Diabetes mellitus | History of diagnosis with type 1 diabetes mellitus or type 2 diabetes mellitus, or hemoglobin A1c greater than 6.5% [[7](#_ENREF_7)]. |
| Functional status | The level of self-care confirmed by the physician on admission to ED, revealing the functional status of patient prior to admitting hospital [[8](#_ENREF_8)].  - Totally dependent: the patient cannot complete any activities of daily living for himself or herself; includes patients who are totally dependent on nursing care (e.g. dependent nursing home patient).  - Partially dependent: the patient needs help from another person for some activities of daily living. Patients admitted from a nursing home setting who are not totally dependent would arrange into this category, as would any patient who requires hemodialysis or home ventilator support yet preserves some independent function.  - Independent: the patient is independent in activities of daily living; includes patient who needs usage of tool(s) or device(s) for independent activities of daily living. |
| Length of stay | Duration of hospitalization. |

**References**

1. Vincent JL, Moreno R, Takala J, Willatts S, De Mendonca A, et al. (1996) The SOFA (Sepsis-related Organ Failure Assessment) score to describe organ dysfunction/failure. On behalf of the Working Group on Sepsis-Related Problems of the European Society of Intensive Care Medicine. Intensive care medicine 22: 707-710.

2. Fine MJ, Stone RA, Singer DE, Coley CM, Marrie TJ, et al. (1999) Processes and outcomes of care for patients with community-acquired pneumonia: results from the Pneumonia Patient Outcomes Research Team (PORT) cohort study. Archives of internal medicine 159: 970-980.

3. Nseir S, Di Pompeo C, Diarra M, Brisson H, Tissier S, et al. (2007) Relationship between immunosuppression and intensive care unit-acquired multidrug-resistant bacteria: a case-control study. Critical care medicine 35: 1318-1323.

4. Arozullah AM, Daley J, Henderson WG, Khuri SF (2000) Multifactorial risk index for predicting postoperative respiratory failure in men after major noncardiac surgery. The National Veterans Administration Surgical Quality Improvement Program. Annals of surgery 232: 242-253.

5. Eric G, Sanjiv C (2009) Diagnostic approach to the patient with cirrhosis. UpToDate, Inc.

6. Dickstein K, Cohen-Solal A, Filippatos G, McMurray JJ, Ponikowski P, et al. (2008) ESC Guidelines for the diagnosis and treatment of acute and chronic heart failure 2008: the Task Force for the Diagnosis and Treatment of Acute and Chronic Heart Failure 2008 of the European Society of Cardiology. Developed in collaboration with the Heart Failure Association of the ESC (HFA) and endorsed by the European Society of Intensive Care Medicine (ESICM). European heart journal 29: 2388-2442.

7. American Diabetes A (2009) Diagnosis and classification of diabetes mellitus. Diabetes care 32 Suppl 1: S62-67.

8. Arozullah AM, Khuri SF, Henderson WG, Daley J, Participants in the National Veterans Affairs Surgical Quality Improvement P (2001) Development and validation of a multifactorial risk index for predicting postoperative pneumonia after major noncardiac surgery. Annals of internal medicine 135: 847-857.
