# Supplementary material for: Prognostic performance of the Rapid Emergency Medicine Score (REMS) and Worthing Physiological Scoring system (WPS) in emergency department
Source: Int J Emerg Med. 2015 Jun 4;8:18. doi: 10.1186/s12245-015-0066-3 (PMC4457731; doi:10.1186/s12245-015-0066-3)
Supplement: Additional file 4: — The Glasgow coma score. [file 12245_2015_66_MOESM4_ESM.docx]

| **Additional file 4**. **The Glasgow coma score (Teasdale and Jennett 1974, cited in [**[**1**](#_ENREF_1)**])** | | |
| --- | --- | --- |
|  |  | Score |
| Eye opening | Spontaneous | 4 |
|  | To speech | 3 |
|  | To pain | 2 |
|  | None | 1 |
| Best verbal response | Oriented | 5 |
|  | Confused conversation | 4 |
|  | Inappropriate words | 3 |
|  | Incomprehensible sounds | 2 |
|  | None | 1 |
| Best motor response | Obeys commands | 6 |
|  | Localizes pain | 5 |
|  | Withdrawal (normal flexion) | 4 |
|  | Abnormal flexion (decorticate) | 3 |
|  | Extension (decerebrate) | 2 |
|  | None | 1 |

**Reference**

1. Sternbach GL (2000) The Glasgow coma scale. The Journal of emergency medicine 19: 67-71.
