# Supplementary material for: Prognostic performance of the Rapid Emergency Medicine Score (REMS) and Worthing Physiological Scoring system (WPS) in emergency department
Source: Int J Emerg Med. 2015 Jun 4;8:18. doi: 10.1186/s12245-015-0066-3 (PMC4457731; doi:10.1186/s12245-015-0066-3)
Supplement: Additional file 5: — The AVPU scale. [file 12245_2015_66_MOESM5_ESM.docx]

| **Additional file 5**. **The AVPU scale (American College of Surgeons 1997, cited in [**[**1**](#_ENREF_1)**]**) | |
| --- | --- |
|  | Level |
| Alert | A |
| Responds to verbal stimuli | V |
| Responds to painful stimuli | P |
| Unresponsive to all stimuli | U |

AVPU scale (A: Alert, V: Verbal, P: Pain, U: Unresponsive).

**Reference**

1. Gill M, Martens K, Lynch EL, Salih A, Green SM (2007) Interrater reliability of 3 simplified neurologic scales applied to adults presenting to the emergency department with altered levels of consciousness. Annals of emergency medicine 49: 403-407, 407 e401.
